# Blood Stream Infections in Burns: A 14-Year Cohort Analysis

**DOI:** 10.3390/life13061357

**Published:** 2023-06-09

**Authors:** Yarden Nitsani, Tal Michael, Dor Halpern, Ariel Avraham Hasidim, Maayan Sher, Rotem Givoli Vilensky, Yuval Krieger, Eldad Silberstein, Yaron Shoham

**Affiliations:** 1 Joyce & Irving Goldman Medical School, Faculty of Health Sciences, Ben-Gurion University of the Negev, Beer-Sheba 8410501, Israel; nitsani@post.bgu.ac.il (Y.N.); dorhal@post.bgu.ac.il (D.H.); arielh@post.bgu.ac.il (A.A.H.); maayas@post.bgu.ac.il (M.S.); 2 Department of Epidemiology, Biostatistics, and Community Health Sciences, Faculty of Health Sciences, Ben-Gurion University of the Negev, Beer-Sheba 8410501, Israel; michtal@post.bgu.ac.il; 3 Clinical Research Center, Soroka University Medical Center, Beer-Sheba 8410101, Israel; givolir@post.bgu.ac.il; 4 Plastic and Reconstructive Surgery Department and Burn Unit, Soroka University Medical Center, Faculty of Health Sciences, Ben-Gurion University of the Negev, Beer-Sheba 8410501, Israel; yuvalkr@bgu.ac.il (Y.K.); eldads@bgu.ac.il (E.S.)

**Keywords:** burn, blood stream infection, bacterial, fungal, outcome

## Abstract

Background: Blood stream infections are a significant cause of morbidity and mortality in burns, and pathogen identification is important for treatment. This study aims to characterize the microbiology of these infections and the association between the infecting pathogen and the hospitalization course. Methods: We conducted a cohort study that included records of burn patients treated at the Soroka University Medical Center between 2007–2020. Statistical analysis of demographic and clinical data was performed to explore relationships between burn characteristics and outcomes. Patients with positive blood cultures were divided into four groups: Gram-positive, Gram-negative, mixed-bacterial, and fungal. Results: Of the 2029 burn patients hospitalized, 11.7% had positive blood cultures. The most common pathogens were Candida and Pseudomonas. We found significant differences in ICU admission, need for surgery, and mortality between the infected and non-infected groups (*p* < 0.001). Pathogen groups differed significantly mean TBSA, ICU admission, need for surgery, and mortality (*p* < 0.001). Multivariate analysis showed flame (OR 2.84) and electric burns (OR 4.58) were independent risk factors for ICU admission and surgical intervention (*p* < 0.001). Gram-negative bacterial infection was found to be an independent predictor of mortality (OR = 9.29, *p* < 0.001). Conclusions: Anticipating specific pathogens which are associated with certain burn characteristics may help guide future therapy.

## 1. Introduction

Burn wound infection is a significant cause of morbidity and mortality in burn patients [1,2]. The burn wound is considered an associated factor in developing bloodstream infections (BSIs), which is a major burn wound complication. The reason lies in the fact that burn wounds are an optimal place for bacterial reproduction and a port of entry to the bloodstream [3]. Besides the wound itself, other causes responsible for BSIs in burn patients are the use of invasive devices and translocation of the gastrointestinal flora [4]. In addition, causative factors for developing serious bacterial infections in children include younger age, the use of a central line, larger total body surface area burned (TBSA%), and ventilator support [5]. Throughout the last decades there have been changes in the incidence of different infecting pathogens; for instance, Singh et al. described a major decrease in the incidence of Gram-positive organisms, when comparing a cohort of burn patients treated during 1997–2002 to a cohort treated during 2010–2014 [6]. Pathogen types in pediatric burn patients may differ from those in adults. In children Gram-positive bacteria were reported as the most common causative agents, followed by Gram-negative bacteria and fungi [4], while in adults Gram-negative infections were more common [7]. The common Gram-negative organisms are *Pseudomonas aeruginosa* (*P. aeruginosa*), *Klebsiella pneumoniae* (*K. pneumoniae*), and *Acinetobacter baumanii* (*A. baumanii*). *Staphylococcus aureus* (*S. aureus*) is the major isolate among Gram-positive organisms [1,6].

Identifying the specific pathogen is important for the management and treatment due to variations in the clinical courses. For example, the median duration of the development of BSI as a result of a Gram-positive infection from the time of burn has been reported to be shorter than that of BSI caused by Gram-negative bacteria and fungal pathogens [4]. This may help to suspect Gram-positive infections in the first few days post-burn. Although topical antimicrobial therapy is used in the prevention of burn wound infections, most authors do not support the use of systemic prophylactic antibiotics when managing burn patients [3,8,9,10]. Antimicrobial resistant bacteria are a major concern due to their clinical and financial impacts. The incidence of multi-drug resistant (MDR) bacteria is on the rise with MRSA being the most common pathogen, followed by MDR *P. aeruginosa* and MDR *A. baumanii* [6]. When considering the choice of treatment with the possible resistances, it is important to acknowledge that prior Meropenem exposure, Gram-negative colonization on admission, escharotomy, and superficial partial-thickness burn size may be potentially important factors for increasing the risk of MDR Gram-negative infection in the critically ill burn patient [11]. Introducing a strategy for burn wound-oriented infection prevention and treatment in burn patients provides various benefits for patients and healthcare systems, including impact on local and global epidemic situations and cost effectiveness [2]. Evaluation of existing protocols and international standards in burn care are necessary to develop standardized guidelines for burn care and to improve the quality of care [12,13].

The aims of this study were to characterize the microbiology of burn wound infections in our medical center, describe the association between the infecting pathogen and the hospitalization course, and characterize the common pathogens groups among burn patients with different burn etiologies.

## 2. Materials and Methods

We conducted a retrospective cohort study that included records of burn patients hospitalized at the Soroka University Medical Center (SUMC, Beer-Sheba, Israel) between January 2007 and December 2020. The study was conducted in accordance with the Declaration of Helsinki and approved by the Institutional Review Board of the Soroka University Medical Center (protocol code 0035-20-SOR, approved 15 June 2020). Patient consent was waived due to the retrospective nature of the study. The inclusion criteria included patients of all ages hospitalized due to isolated burn injuries. Exclusion criteria were patients suffering from cold injuries, friction injuries, multi-trauma and patients with missing data on medical records.

Epidemiologic and demographic data were collected retrospectively, as well as clinical variables. Patients with 1 or more positive blood cultures during their hospitalization period were divided into 4 groups according to the infecting pathogens. The Gram-positive group included patients with 1 or more cultures positive for only Gram-positive bacteria. The Gram-negative group included patients with 1 or more cultures positive for only Gram-negative bacteria. The mixed bacterial group included patients with a combination of 1 or more cultures positive for both Gram-positive bacteria and Gram-negative bacteria, either in the same culture or in more than 1 culture. The fungal group included patients with 1 or more cultures positive for fungal pathogens regardless of whether they had any cultures positive for any bacterial growth.

The endpoints of this study were to characterize the different blood culture groups (age, gender, burn etiology, burn area) and to compare patient outcome measures as a function of blood culture grouping. Patient outcome measures included hospital length of stay, ICU admission and length of stay, need for surgical intervention and number of surgical interventions, and mortality. Criteria for ICU admission in our center include major burn injuries (i.e., adult patients with more than 15% TBSA involvement and more than 10% for children or elderly patients), inhalation injury with airway involvement, or hemodynamic instability.

Blood cultures were obtained at the discretion of the treating physicians; however, in general, indications for ordering blood cultures in our center include a fever >38.5 °C, a rapidly descending WBC count or leukopenia <4.5 × 10^3^/µL, a lactate ≥ 2.0 mg/dL or hemodynamic instability. Antibiotics were administered after obtaining blood cultures to prevent false-negative results.

### Statistical Analysis

Dichotomous and nominal variables are described using mode; numeric variables are described using mean as a central tendency measure, and standard deviation as a dispersion measure. The associations between different outcomes and independent variables were tested using different statistical tests. Among our anticipated outcomes, two main types of variables were found: nominal (e.g., death during/after hospitalization), or time to events, hence the different tests related to each variable type. The association between the nominal outcome to nominal independent variables, such as gender or cause of burn, was tested using the Chi-square test. The association between the outcome and continuous variables, such as age, was tested using a *t*-test and Mann–Whitney tests. As for the chronological variables, such as days to complete wound closure or other periods before events prior to undergoing a survival analysis, Kaplan–Meyer charts were examined, log-rank tests were performed, and the proportional hazard assumption was tested for each couple of time-dependent variables, and every independent variable. Variables that were found to be associated with the dependent variables (*p*-value < 0.1) were gradually included in the final models. The final analysis included logistic models and cox-regression models. Significance was examined in two-tail hypothesis tests and significance was defined as a *p*-value < 0.05. Statistical analysis was performed using SPSS (version 25.0, IBM) and “R statistics” (R Core Team, Version 4.2.0).

## 3. Results

During the years 2007–2020, a total of 2029 eligible burn patients were admitted to the Soroka University Medical Center: 613 adults and 1416 children. The study sample was composed of 1340 males (66%) and 689 females (34%), and patients’ ages ranged from 0 to 93 years. The most common cause of burn was scald (1083 patients, 53.4%), followed by flame burns (541 patients, 26.7%), chemical burns (187, 9.2%), electric injury (69 patients, 3.4%), and other causes including steam, hot gas, contact and oil burns (146 patients, 7.2%). Demographic and clinical data of the pediatric and adult populations are shown in Table 1, and data divided by blood culture results are shown in Table 2.

**Figure 1:**
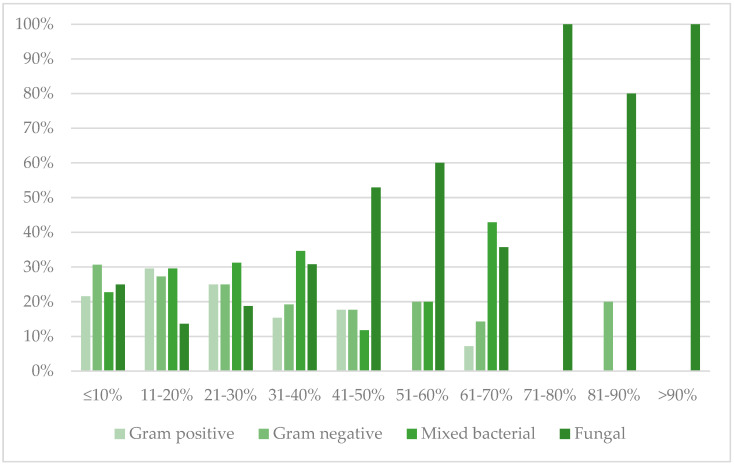
Pathogen group distribution by TBSA.

TBSA was divided into ten groups of 10% intervals (e.g., 1–10%, 11–20%). The area of most burns (73.47%) was <10% TBSA and the interval of pathogen frequencies is shown in Figure 1.

**Figure 2:**
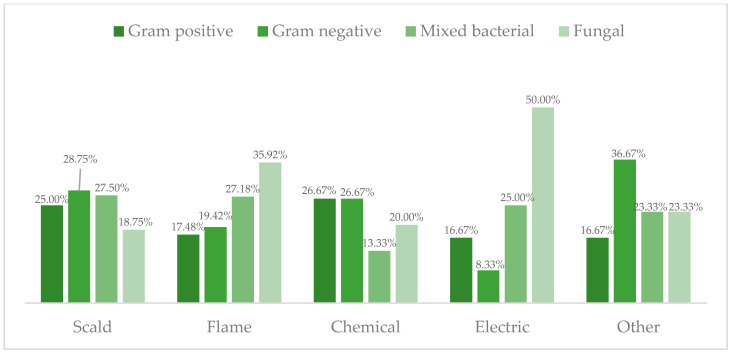
Frequencies of specific pathogens by cause of burn in all ages.

In 88.3% of the cases, blood cultures were negative. Among the 11.7% of patients with positive blood cultures, 24.79% were infected with only Gram-negative bacteria, 20.59% were infected with only Gram-positive bacteria, 26.05% were infected with mixed bacterial infections, and 28.57% were co-infected with fungi. The frequencies of the pathogen groups according to burn etiology are presented in Figure 2.

**Figure 3:**
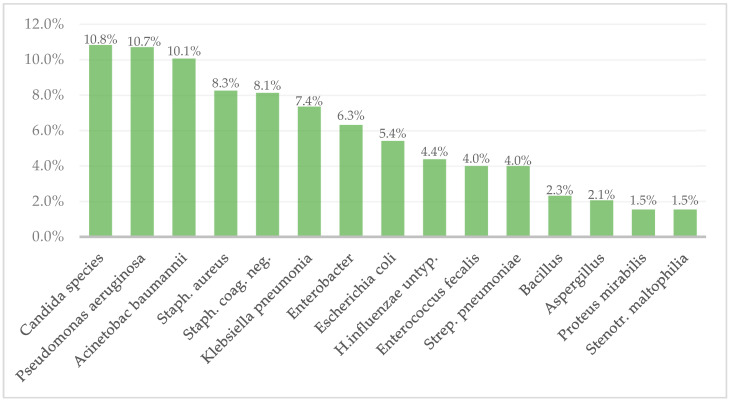
Pathogen frequency in positive cultures.

The most common isolate was Candida species with 10.83% and *P. aeruginosa* with 10.7% of all positive cultures. Additional common pathogens were Acinetobacter species (10.06%), *S. aureus* (8.25%), Klebsiella species (7.35%), Enterobacter species (6.32%), and *Escherichia coli* (5.4%). Specific pathogen growth distributions are seen in Figure 3.

Of all patients, 238 (11.7) had positive blood cultures with a mean age of 21 years old, of which 70% were male. Of the infected pediatric burn patients, 43% were caused by flame, and 33% were caused by scald. The mean TBSA of the infected group was 28.6%. The hospitalization duration of patients with positive blood culture was between 0–139 days with a median of 7 days. In addition, 95 (40%) of them were admitted to the ICU, with a median length of stay of 9 days. A total of 35% of infected patients needed surgery, with a median of two surgeries. The mortality of the infected patients was 10%, as compared to 0.9% among non-infected burn patients (*p* ≤ 0.001). 

When comparing non-infected and infected burns, we found significant differences in age (12 ± 17 vs. 21 ± 20 years, *p* < 0.001), burn etiology (*p* < 0.001), TBSA (13.2% ± 8.19 vs. 28.6% ± 21.74, *p* < 0.001), days of hospitalization (4 ± 128 vs. 7 ± 139, *p* < 0.001), admission to ICU (11% vs. 40%, *p* < 0.001), need of surgical intervention (14% vs. 35%, *p* < 0.001), and mortality (0.9% vs. 10%, *p* < 0.001). There were no differences between the groups in gender (male 65% vs. 70%, *p* = 0.115).

The average time between hospital admission and first positive blood culture was 4.7 ± 2.7 days for the Gram-positive group, 6.9 ± 3.5 days for the Gram-negative group, 5.1 ± 2.5 days for the mixed bacterial group, and 4.4 ± 2.5 days for the fungal group. The only difference between the groups found to be statistically significant was between the Gram-negative group and the fungal group (*p* < 0.01).

### 3.1. Pediatric Population

The pediatric population consisted of 1416 patients between the ages of 0–18 years, comprising approximately 70% of all hospitalized burn patients in the study. Demographic and clinical data of pediatric patients with positive blood culture divided by pathogen groups are presented in Table 3a.

Of all positive cultures among the pediatric population, most had mixed bacterial growth (29%) followed by fungi (26%), Gram-negative (23%), and Gram-positive pathogens (22%). Age (*p* < 0.001) and cause of burn (*p* < 0.001) were significantly different between the different groups of infectious pathogens, while gender did not differ between the groups (*p* = 0.34). TBSA differed between pathogenic groups (*p* < 0.001) and pairwise comparison showed a significant difference between non-infected and infected groups, i.e., Gram-positive bacteria (*p* < 0.001), Gram-negative (*p* < 0.001), mixed bacterial (*p* < 0.001) and fungal infections (*p* < 0.001). There was a significant difference between pathogen groups and median length of hospitalization (*p* < 0.01). Pathogenic groups differed in admission to ICU (*p* < 0.001) and length of stay in ICU (*p* < 0.01), need for surgical interventions (*p* < 0.001), number of surgeries (*p* < 0.01), and mortality (*p* < 0.001).

### 3.2. Adult Population

The adult population included 613 patients aged 18–93 years old, and 109 (17.8%) of these patients had positive blood cultures. Demographic and clinical data of adult patients with positive blood culture divided by pathogen groups are presented in Table 3b.

Out of all positive cultures among the adult patients, most were with fungal growth (32%) followed by Gram-negative (27%), mixed bacterial (22%), and Gram-positive infections (19%). Gender (*p* = 0.03) and cause of burn (*p* > 0.01) were significantly different between groups of infectious pathogens, while median length of hospitalization (*p* = 0.187) and age (*p* = 0.167) did not differ between the groups. TBSA differed between pathogenic groups (*p* < 0.001) and pairwise comparison showed a significant difference between non-infected and infected groups, i.e., Gram-positive bacteria (*p* < 0.001), Gram-negative (*p* = 0.01), mixed bacterial (*p* < 0.001) and fungal infections (*p* < 0.001). Moreover, TBSA in the pairwise comparison showed a significant difference between Gram-negative and fungal infections (19.7% ± 11.1 vs. 47.1% ± 30.8, *p* = 0.02). Pathogenic groups differed in admission to ICU (*p* < 0.001) and median length of stay in ICU (*p* = 0.03), need for surgical interventions (*p* < 0.001), number of surgeries (*p* < 0.01), and mortality (*p* < 0.001)

### 3.3. Multivariate Analysis

ROC curves were used to assess the ability to use the pathogen group as a predictor of certain outcomes. The area under the curve (AUC) for ICU admission was 0.62 (SD 0.02, 95% CI 0.58–0.66, *p* < 0.001), the need for surgical intervention, the AUC was 0.58 (SD 0.01, 95% CI 0.56–0.61, *p* < 0.001), and for mortality, the AUC was 0.76 (SD 0.04, 95% CI 0.67–0.85, *p* < 0.001).

Using logistic regression, we analyzed the relationship between age, gender, burn etiology, TBSA, and the pathogen groups, with the following dependent variables; ICU admission, need for surgery, and mortality. Gram-negative infection was found to be a significant predictor of mortality (Table 4).

The variables that were independently associated with an increased risk of admission to ICU were burn etiology, namely flame burns (OR 2.84; 95% CI 1.29–6.24, *p* < 0.01) and electric injury (OR 4.58; 95% CI 1.70–12.29, *p* < 0.01). The same model was also found significant in predicting the need for surgical intervention (*p* < 0.001). Scald etiology was a negative predictor (OR 0.29; 95% CI 0.17–0.49 *p* < 0.001), whereas flash-burns had a higher risk (OR 6.56; 95% CI 1.83–23.45, *p* < 0.01). Using these variables, we found that a positive blood culture did not predict ICU admission or need for surgery, while standardized to gender, age, cause of burn, and TBSA; however, we found Gram-negative bacteria infection to predict mortality (OR = 9.29; 95% CI 1.79–48.03, *p* < 0.001).

## 4. Discussion

In this study, we evaluated the hospitalization course of infected burn patients. We conducted a study on a large and diverse cohort and show our results divided into two main populations—children and adults. Most of our patients were children (70%), and the majority of patients had TBSA under 10%, similar to other studies [14]. In our study, we considered only positive blood cultures as a significant sign of infection, as this is recognized as a main reason for morbidity and mortality among burn patients [15]. We found that 11% of all our burn patients (mean TBSA = 15%) developed bloodstream infections. There is a wide range of positive blood culture rates reported in the literature. For example, Patel et al. [16] reported only a 4% rate of positive blood cultures in their cohort (mean TBSA = 40%), and Bahemia et al. [17] reported a 52% rate in their cohort (mean TBSA = 31%). More common reported ranges include the following analyses: Song et al. reported 1.1% positive blood cultures for patients with TBSA < 20% and 18.6% positive blood cultures for patients with TBSA ≥ 20% [18]; Hu et al. reported a 27.5% positive blood culture rate for patients with TBSA > 20% [19]; Karimi et al. reported a 12% positive blood count rate in a cohort with a mean TBSA of 21.6% [20]; Lee et al. reported a 17% positive blood culture rate in a cohort with a mean TBSA of 49% [21]; and Latifi et al. reported a 7.6% rate of positive blood cultures in a cohort with a mean TBSA of 16% [22]. Thus, we believe the rate found in our study is within the more common ranges reported in the literature, based on the average burn area of our cohort. We assume the differences in developing BSI depend on the depth and burn area in different studies, as well as different infection prevention regulations, timing of antibiotic administration and the existence of dedicated burn intensive care units.

The 239 patients with positive blood cultures had 775 positive blood cultures, and the most common pathogens were Candida (*n* = 84, 10.8%) and Pseudomonas species (*n* = 83, 10.7%), followed by Acinetobacter species (*n* = 78, 10.1%) and S.aureus (*n* = 64, 8.3%). Our findings are mostly in line with the literature. Kaita et al. reported that the most frequently isolated organisms in their study were fungi, specifically candida species [23]. Brusselaers et al. reported that 13.2% of positive blood cultures were positive for Candida [24]. In fact, fungal infection rates (mostly Candida spp.) in burn patients are quite high, with most reports ranging from 6.3% to 15%, although there are significant differences between individual burn centers ranging from 0.7% up to 24.1% [25,26,27,28,29,30,31]. Yin et al. evaluated only bacterial infections and found the highest frequencies were *K. pneumoniae*, *P. aeruginosa*, and *A. baumannii* [32]. Additional studies reported that Gram-positive pathogens were more commonly associated with BSI than Gram-negative pathogens [33,34]. In addition, we expected MRSA to grow in higher frequencies, as was described in previous studies [16,19]; however, our study showed only one MRSA growth. Inconsistencies are optional due to changes in different environments and monitoring programs in every burn center.

We found that the most common burn etiology in infected patients was scalds in children (51.9%) and flame burns in adults (54.1%). These findings are also in line with the literature. Several studies [14,35,36] reported scald to be the most common cause of burns among pediatric patients and Wang et al. [37] found flame to be the most common cause of burns among adult patients above the age of 15 years old. Different etiologies can derive from an awareness level change in different ages, and also different daily schedules in all ages; while most pediatric burns happen at home, occupational risk is a concern in the adult community [36].

When comparing and evaluating pathogen group frequencies in different burn etiologies, we found the most common infection group in both scald and flame burns was fungal. Data on analyzing the connection between specific pathogens and burn etiology is limited; therefore, we believe further studies assessing this specific connection may be beneficial in terms of patient care and hospital resources.

We found higher infection rates were associated with flame burns and larger TBSA among all ages. We assume this is connected to the fact that more severe damage to the skin, the natural barrier against infections, leads to invasive infections and lowers the immune system ability to fight pathogens.

Our study shows that infected patients in all age groups had worse outcomes compared to non-infected patients. BSI were associated with longer hospitalizations, more need for surgical intervention, and mortality. These findings are also in line with previously published data [15]. More specifically, the fungal infection group in all ages contained higher rates of flame burns relative to the other pathogen groups. Moreover, the mean TBSA of the fungal infected burn patients was the highest, followed by mixed bacterial Gram-positive and Gram-negative bacteria. These results are expected, as flame burns tend to relate to more extensive burn areas [38]. We also found higher rates of fungal infections in electric burns among children, possibly because they involve multi-organ damage and put a high-systemic demand on the patient’s immune system. Patients with fungal infections had worse outcomes than those infected by other pathogenic groups in terms of the variables mentioned above (ICU admission, surgeries, and mortality).

Using logistic regression, we evaluated the relation of clinical variables with clinical outcomes. After standardizing for age, gender, TBSA, and pathogen group, we found a significant correlation between ICU admission and flame and electric burns. These burn etiologies are usually associated with more extensive and deeper burns, which also affect numerous systems such as cardiovascular and respiratory; thus, the necessity for monitoring is crucial [33,39]. Gram-negative bacteremia was found to be a mortality predictor when standardizing for all variables mentioned above. A possible explanation is that in our study, Gram-negative bacteria tended to infect less extensive minor scald burns that have overall lower mortality rates. The fact that the fungal infection group was not found to be an independent contributor to mortality may be explained by the fact they were associated with more extensive flame burns, which are themselves major mortality predictors.

With regard to the average time from hospital admission to the first positive blood culture, we found the range in all groups was between 4.4 to 6.9 days. These findings are in line with Raz-Pastour et al., who reported that the majority of first BSI episodes were diagnosed during the first week of hospitalization [40]. Additionally, we found that the average time to first positive blood culture of the Gram-negative group (6.9 days) was significantly longer than that of the fungal group (4.4 days); however, we did not find any other significant differences between the groups. Therefore, we cannot state that the time to first positive blood culture of any specific pathogen group is significantly different than all the other pathogen groups. Standard practice in our center is in line with the current literature that does not support the role of systemic antibiotic prophylaxis in the management of burn patients [3,9,10]. Based on the results of our study we do not see a reason to recommend otherwise.

Our study has several limitations. Firstly, our Burns Unit is the only one in southern Israel, treating a population of nearly 1.5 million inhabitants. Due to the location of our center and the nature of our population, nearly all burn patients in southern Israel are admitted to our center within 24 h of injury. Therefore, it is reasonable to assume nearly none arrived at our center already infected. Nevertheless, we recognize that including patients regardless of their time from injury to admission may be one of our study’s limitations. Additionally, our study was a retrospective, single-center study, with a relatively small positive culture sample size. Therefore, our results and conclusions may imply to our population and setting. Another study limitation is some degree of missing data we encountered in the electronic medical records, which somewhat limited our analysis. Additionally, we recognize that the grouping of the fungal group is not ideal because the contribution of bacterial infections in this group is unclear. Nevertheless, we found that the vast majority of the fungal positive culture patients also had positive bacterial cultures; therefore, we did not further separate this group.

## 5. Conclusions

This study demonstrates that fungal infections were associated with worse outcomes in terms of ICU admission, need for surgical intervention, and mortality. When cancelling confounders such as age, burn etiology, and burn area, a Gram-negative blood stream infection was found to be an independent predictor of mortality. These findings may suggest that a special focus on prevention and prophylaxis in major burns should address these pathogens. In smaller, less significant burns, Gram-negative bacterial infections should be taken seriously because they may complicate these patients’ otherwise benign clinical course. We recommend further research focusing on more specific pathogen groups and the association between them and patient outcomes. Anticipating specific pathogen association with certain burn characteristics may better guide future therapy approaches and help clinicians find the most appropriate approach to prevent complications.

## Figures and Tables

**Table 1:** Demographic and clinical data of 2029 burn patients.

	Total 2029	Pediatric 1416	Adult 613	*p*-Value
Male gender N (%)	1340 (66%)	878 (62%)	466 (76%)	<0.001
Cause of burn:				
Scald (%)	53.4%	67%	22.2%	<0.001
Flame (%)	26.7%	20%	42.3%	<0.001
Chemical (%)	9.2%	4.6%	19.9%	<0.001
Electric (%)	3.4%	3%	4.4%	<0.001
Other (%)	7.2%	5.6%	10.9%	<0.001
TBSA mean (±SD)	15% (11.7)	13.9% (8.9)	17.7% (16.2)	<0.001
Positive blood culture	237 (11.7%)	129 (9.1)	109 (17.8)	<0.001
Admission to ICU	292 (14.4%)	171 (12.1%)	121 (19.7)	<0.001
Need of surgery	16.6%	12.1%	26.8%	<0.001
Mortality	2.1%	0.6%	5.5%	<0.001

**Table 2:** Demographic and clinical data of 2029 burn patients divided by blood culture results.

	No Growth	Positive Culture	Statistic	*p*-Value
Number of patients N (%)	1791 (88.3%)	238 (11.7%)	-	
Age (years) mean (±SD)	12 (17)	21 (20)	T = 6.8	<0.001
Male gender (%)	65%	70%	CS = 2.48	0.115
Cause of burn:			CS = 64.79	<0.001
Scald (%)	56%	33%		
Flame (%)	24%	43%		
Chemical (%)	10%	5.5%		
Electric (%)	3%	5%		
Other (%)	6.6%	12%		
TBSA mean (±SD)	13.2% (8.19)	28.6% (21.74)	MW, U = 416,038.5	<0.001
Days of hospitalization	4 (128)	7 (139)	MW, U = 276,155.5	<0.001
Admission to ICU	11%	40%	CS = 11.12	<0.001
ICU days median (range)	3 (95)	9 (72)	MW, U = 12,482.5	<0.001
Need of surgery (%)	14%	35%	CS = 71.59	
Surgeries mean (±SD)	2.23% (1.53)	2.98 (1.77)	MW, U = 19,247	
Mortality (%)	0.9%	10%	CS = 104.27	<0.001

MW—Mann–Whitney; CS—Chi-square.

**Table 3:** (**a**) Demographic and clinical data of pediatric burn patients divided by pathogen groups. (**b**) Demographic and clinical data of adult burn patients divided by pathogen groups.

**(a)**							
	**No Growth**	**Gram** **Positive **	**Gram** **Negative**	**Mixed** **Bacterial**	**Fungal**	**Statistic**	***p*** **-Value**
Number of patients N (%)	1287 (90.8%)	28 (1.9%)	30 (2.1%)	38 (2.6%)	33 (2.3%)	-	-
Age (years) mean (±SD)	3.5 (18)	4.5 (4.6)	6.4 (5.1)	5.1 (5.6)	5.8 (4.7)	ANOVA = 484.27	<0.001
Male gender (%)	61.1%	53%	73%	71%	66%	CS = 4.45	0.34
Cause of burn:						CS = 44.12	<0.001
Scald (%)	68.4%	60.7%	53.3%	55.3%	39.4%		
Flame (%)	18.5%	32.1%	30%	31.6%	42.4%		
Chemical (%)	5%	3.6%	-	-	-		
Electric (%)	3%	-	-	2.6%	9.1%		
Other (%)	5.1%	3.6%	16.7%	10.5%	9.1%		
TBSA mean (±SD)	12.7% (6.1)	19.6% (12)	27.3% (22.4)	26.1% (17)	29.1% (21.5)	KW = 129.84	<0.001
Days of hospitalization median (range)	20 (152)	40 (59)	48 (143)	25 (118)	31 (199)	KW = 13.89	<0.01
Admission to ICU	9.4%	32.1%	36.7%	44.7%	42.4%	CS = 102.65	<0.001
ICU days median (range)	3 (53)	10 (21)	11 (66)	7 (50)	10.5 (67)	KW = 17.27	<0.01
Need of surgery (%)	10.3%	21.4%	33.3%	36.8%	27.3%	CS = 47.63	<0.001
Surgeries mean (±SD)	2.1 (1.6)	3.33 (1.63)	2.4 (1.07)	2.36 (0.63)	3.11 (2.02)	KW = 13.78	<0.01
Mortality (%)	0.2%	7.1%	-	2.6%	6.1%	CS = 44.89	<0.001
**(b)**							
	**No Growth**	**Gram** **Positive**	**Gram** **Negative**	**Mixed** **Bacterial**	**Fungal**	**Statistic**	***p*** **-Value**
Number of patients N (%)	504 (82.2%)	21 (3.4%)	29 (4.7%)	24 (3.9%)	35 (5.7%)		-
Age (years) mean (±SD)	36.5 (75)	37 (15)	40 (16.5)	37 (49)	43 (70)	ANOVA = 1648.8	0.167
Male gender (%)	76%	71.4%	58.6%	95.8%	77.1%	CS = 10.40	0.03
Cause of burn:						CS = 42	<0.01
Scald (%)	24.4%	14.3%	24.1%	4.2%	5.7%		
Flame (%)	39.7%	42.9%	37.9%	66.7%	65.7%		
Chemical (%)	21.8%	14.3%	13.8%	8.3%	8.6%		
Electric (%)	3.8%	9.5%	3.4%	8.3%	8.6%		
Other (%)	9.9%	19%	20.7%	12.5%	11.5%		
TBSA mean (±SD)	14.6% (11.8)	26% (16)	19.7% (11.1)	30.9 (21.5)	47.1% (30.8)	KW = 102.24	<0.001
Days of hospitalization median (range)	17 (223)	61.5 (72)	12.5 (169)	34.5 (95)	39.5 (102)	KW = 6.16	0.187
Admission to ICU	14.9%	28.6%	34.5%	41.7%	42.9%	CS = 50.71	<0.001
ICU days median (range)	2 (95)	25.5 (36)	1.5 (72)	15 (45)	8 (51)	KW = 10.07	0.03
Need of surgery (%)	23.4%	47.6%	27.6%	45.8%	48.6%	CS = 20.50	<0.001
Surgeries mean (±SD)	2.34 (1.44)	3.2 (2.89)	2.63 (1.06)	3.45 (2.65)	3.35 (1.45)	KW = 17.17	<0.01
Mortality (%)	2.6%	19%	3.4%	16.7%	34.3%	CS = 76.86	<0.001

KW—Kruskal–Wallis; CS—Chi-square.

**Table 4:** Multivariate survival analysis—Cox regression.

Mortality								
	B	S.E.	Wald	df	Sig.	Exp (B)	95% C.I. for EXP (B)	
							Upper	Lower
Positive culture			12.45	4	0.01			
Gram-positive	0.02	0.65	0.001	1	0.97	1.025	0.286	3.669
Gram-negative	2.23	0.84	7.074	1	0.01	9.292	1.798	48.033
Mixed-Bacterial	−1.48	1.21	1.5	1	0.22	0.228	0.021	2.431
Fungal	0.14	0..85	0.025	1	0.87	1.146	0..215	6.11

Variables: culture, age, gender, burn etiology, TBSA.

## Data Availability

The data presented in this study are available on request from the corresponding author. The data are not publicly available due to privacy restrictions.
